# SOD1 stimulates lamellipodial protrusions in Neuro 2A cell lines

**DOI:** 10.1080/19420889.2018.1486652

**Published:** 2018-08-09

**Authors:** Ilaria Ferrari, Chiara Verpelli, Arianna Crespi, Carlo Sala, Diego Fornasari, Grazia Pietrini

**Affiliations:** a Department of Medical Biotechnology and Translational Medicine, Università degli Studi di Milano, Milan, Italy; b Institute of Neuroscience, Consiglio Nazionale delle Ricerche, Milan, Italy

**Keywords:** Amyotrophic lateral Sclerosis (ALS), Insulin receptor substrate of 53 kDa (IRSp53), Lamellipodia, LIN7, Rac1 GTPases, Superoxide dismutase 1 (SOD1), SOD1 mutants G93A and G147S

## Abstract

We here investigated the effects of overexpressed superoxide dismutase (SOD)1 and amyotrophic lateral sclerosis (ALS)-linked SOD1 mutants G93A and G147S in Neuro 2A (N2A) cell lines, and found a three-fold increase in lamellipodia either in cells cultured under differentiated or undifferentiated growth conditions. In undifferentiated N2A cells, SOD1 constructs promoted lamellipodial protrusions to similar extent as the overexpression of Rac1, and SOD1-mediated lamellipodia were prevented by coexpression of the N17 dominant-negative form of Rac1, or shRNA for a downstream effector of Rac1, the insulin receptor tyrosine kinase substrate p53 (IRSp53) or its binding partner LIN7. Moreover, no additive effect was measured by coexpression of the SOD1 constructs with Rac1, IRSp53 or LIN7. Collectively these data support a role for SOD1 in the regulation of Rac1-mediated lamellipodia pathway, a property fully retained by the two SOD1 mutants.

## Introduction

Amyotrophic lateral sclerosis (ALS) is a devastating age-related neurodegenerative disease characterised by a progressive loss of motor neurons of the central nervous system, leading to progressive muscle denervation and paralysis [1]. No effective therapy is available for ALS, and understanding the disease pathogenesis could help in developing effective treatments. The majority of ALS cases are sporadic while familial forms account only for 10% of all ALS [2]. Twenty percent of inherited ALS is caused by mutations in the gene encoding for superoxide dismutase 1 (SOD1), an ubiquitously expressed enzyme functioning in the clearance of potentially toxic superoxide radicals. The resulting mutant proteins have additional but still unclear functions that are toxic for motor neurons. A non-cell autonomous mechanism involving contiguous and functionally related glial cells has also been implicated in the disease [3]. Whereas damage to motor neurons is associated to the onset of ALS, damage to astrocytes and microglia severely accelerates disease progression [4–6].

Toxicity of mutant SOD1 has been associated with overactivation of NADPH oxidase (NOX2), due to an apparent higher affinity of mutants to the Rac1 member of the Rho GTPase family, compared to the wild-type (WT) SOD1, which in turn would lock NOX2 in its active superoxide-producing form even in oxidising conditions when the WT form dissociates from Rac1 [7].

Rac1 is, however, known to play a major role in the regulation of the actin cytoskeleton inside lamellipodia, the actin-based membranous projections that usually appear on moving edge of any cell type [8] and that are fundamental for neurite formation and collateral branches outgrowth [9]. We therefore analysed whether SOD1 may promote lamellipodial protrusions, and whether this property is retained by ALS-associated mutated SOD1. The SOD1 mutants used in this study are the well characterised G93A mutant, which is known to retain the dismutase catalytic activity of the WT enzyme [10], and the less studied G147S, lacking of the catalytic activity and causing a very rapid clinical course of the disease [11]. The effects of SOD1 overexpression were investigated in Neuro 2A (N2A) cells, a mouse neural crest-derived cell line that exhibit fibroblast-like morphology when cultured in regular medium supplemented with 15% serum, while differentiate into neuronal-like cells within a few days when cultured in serum-deprived medium [12].

The effects of SOD1 overexpression on lamellipodia formation were quantitatively analysed by phalloidin immunostaining to visualise the typical F-actin staining inside lamellipodia. The SOD1-mediated pathway for lamellipodia was investigated by coexpression experiments with WT or a dominant negative Rac1, and by interfering with the expression of a downstream effector of Rac1, the insulin receptor substrate of 53 kDa, IRSp53 [8,13], and its binding partner LIN7 [14]. In addition, to verify the effect of SOD1 overexpression in neuronal differentiation, the lamellipodia promoting activity of SOD1 constructs was also investigated in N2A cells cultured under serum-free conditions.

## Results

### Overexpression of SOD1 mediates Rac1-dependent lamellipodia extension in neuronal-like N2A cells

To test whether SOD1 could induce the formation of membrane protrusions in neuronal cells, we ectopically overexpressed untagged WT human SOD1 or the ALS-linked G93A and G147S mutants in N2A cells. Approximately a 8 fold level of expression over the endogenous was measured for each transfected SOD1 (see Figure S1). In addition, as negative and positive controls, the cells were transiently transfected with GFP fused to a plasma membrane localisation signal (mGFP) and Rac1 fused to HA, respectively. Cells cultured in undifferentiated growth conditions (15% FBS) for 48 hours after transfection were scored for the presence of lamellipodial protrusions by phalloidin staining of F-actin.

**Figure 1.**
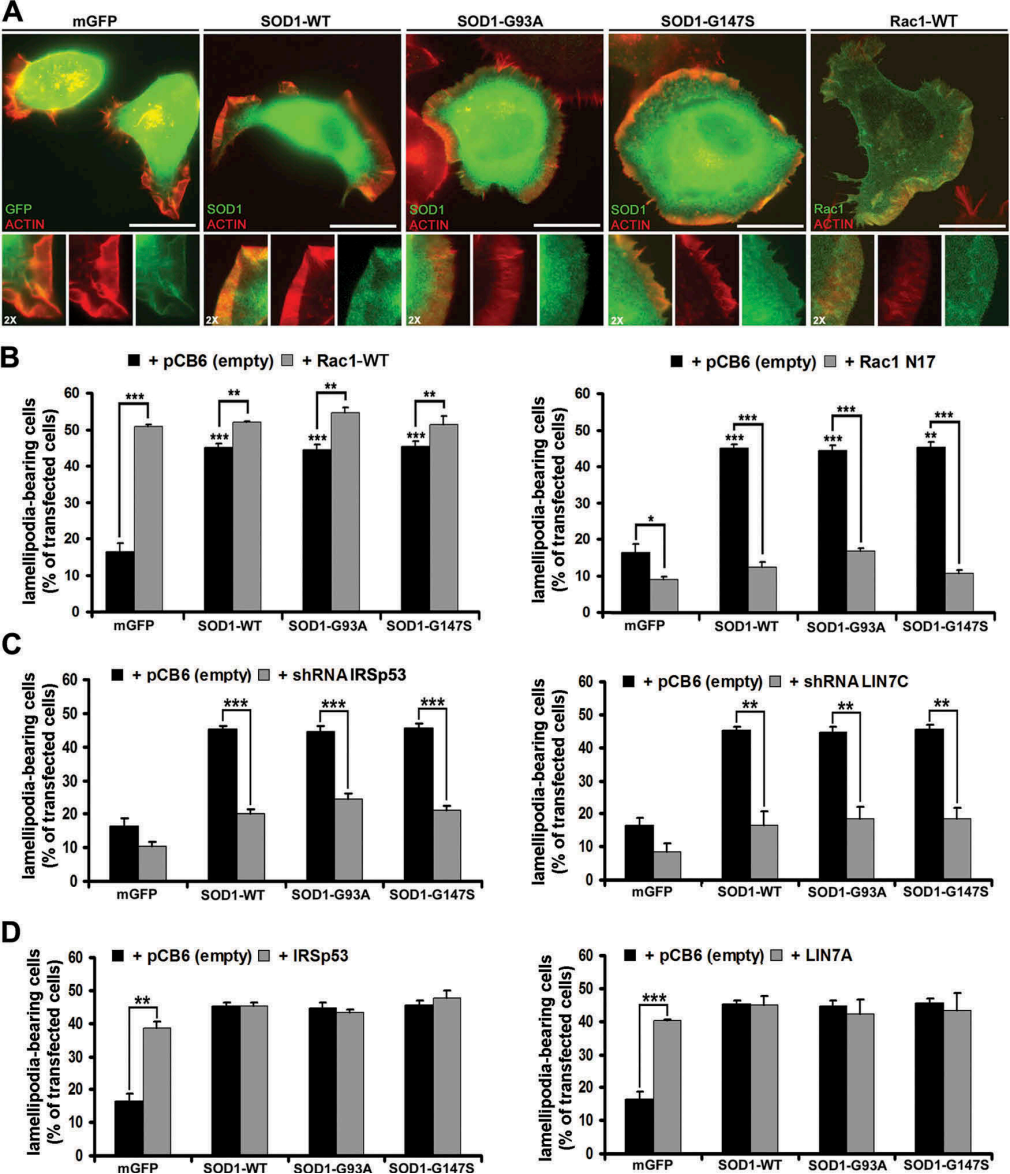
Wild-type SOD1 and ALS-linked SOD1 G93A and G147S mutants induce Rac1-dependent extension of lamellipodia in N2A cells cultured under undifferentiated conditions. (A) Wide-field microscopy of N2A cells transfected with the indicated cDNA. Twenty-four hours after transfection, cells were fixed in 4% paraformaldehyde for 20 min at 37°C and stained with Rhodamine-conjugated Phalloidin to visualise F-actin enriched lamellipodia (red), and anti-SOD1 or anti HA-tag to localise Rac1 (green). A 2X individual staining magnification of lamellipodia is shown. Scale bar; 10 μm. (B-D) Histograms representing quantifications of the percentage of the transfected cells exhibiting lamellipodia, 24 hours after cotransfection with the indicated cDNAs (n > 120 cells from 3 independent experiments). Error bars indicate ± s.e.m.; P-values (t-test): *P < 0.05, **P < 0.01 and ***P < 0.001  indicate significance between mGFP and WT SOD1 or between mGFP and SOD1 mutants.

In these culture conditions, N2A cells have either a spherical or fibroblast-like morphology, and extend lamellipodia and filopodia in order to sense and sample the surrounding environment. Localisation of SOD1 as well as Rac1 in F-actin enriched lamellipodia was clearly revealed by indirect double immunofluorescence staining with the specific antibodies (Figure 1(A)).

We next compared the lamellipodia-inducing capacity of SOD1 with that of Rac1, and analysed the effect of their cotransfection (Figure 1(B)). Approximately a three-fold increase in the percentage of lamellipodia-bearing cells was found in Rac1-transfected cells (~52%) compared to mGFP transfected cells (~17%). The overexpression of SOD1, either WT or mutants, promoted a similar increase in lamellipodia than Rac1 (~47%). The same effect measured in cells expressing Rac1 alone was obtained in cells coexpressing Rac1 with each of the SOD1 constructs (~52%), suggesting that SOD1 and Rac1 are in the same pathway.

In order to verify this possibility, Rac1 was inhibited by expression of a dominant negative mutant, the HA-tagged Rac1-N17 (Figure 1(B)). As expected, inhibition of Rac1 activity decreased the number of cells with lamellipodia under 10% in control mGFP-cells, and, most importantly, the percentage of lamellipodia-bearing cells remained at control levels also in SOD1-cotransfected cells (~13,5%), thus suggesting that SOD1 is unable to stimulate lamellipodia when Rac1 activity is inhibited.

In line with data in literature indicating a main role for Rac1 activation in the formation and function of lamellipodia rather than filopodia, we did not detect any increase in filopodia in cells expressing SOD1 and/or Rac1 (data not shown).

### The IRSp53-LIN7 complex is a downstream effector of SOD1-mediated extension of lamellipodia

We next tested SOD1 involvement in Rac1–mediated pathway for lamellipodia formation by altering the expression of a downstream effector of Rac1, the insulin receptor substrate protein of 53 KDa (IRSp53, also named BAIAP2), which acts as a link between Rac1 and WAVE-ARP 2/3 complexes in signalling lamellipodia formation [8,13].

The IRSp53 protein contains multiple domains for protein–protein interactions, including a PDZ domain-binding motif at its C-terminus for interaction with the scaffold protein LIN7 (also named MALS/Velis), and LIN7 association is required to positively regulate IRSp53 activity at specific membrane sites in epithelial and neuronal cells [14–17]. We therefore tested whether IRSp53 and LIN7 play a role in SOD1-dependent formation of lamellipodia by analysing the percentage of lamellipodia-bearing cells in SOD1 overexpressing N2A cells downregulated for/coexpressing IRSp53 or LIN7 (Figure 1(C–D)).

SOD1-mediated increase in lamellipodia was greatly prevented in N2A cells downregulated for IRSp53 or LIN7C, the LIN7 form mainly expressed by these cells [16]. In line with a role for the IRSp53-LIN7 complex in lamellipodia formation, the percentage of SOD1-expressing cells with lamellipodia (~45%) dropped to ~21% and ~17% in cells downregulated for IRSp53 or LIN7, respectively. Moreover, the overexpression of IRSp53 or LIN7 promoted a > two-fold increase in the percentage of cells with lamellipodia (from 17% to ~40%), and no further increase was observed in cells co-expressing IRSp53 or LIN7 with any of the SOD1 constructs (Figure 1(D)).

Once again we did not observe a different behaviour among SOD1 WT and the mutants, thus indicating that these mutants retain their function in promoting lamellipodia.

### Overexpression of SOD1 stimulates formation of lamellipodia in N2A cells cultured under differentiated growth conditions

**Figure 2.**
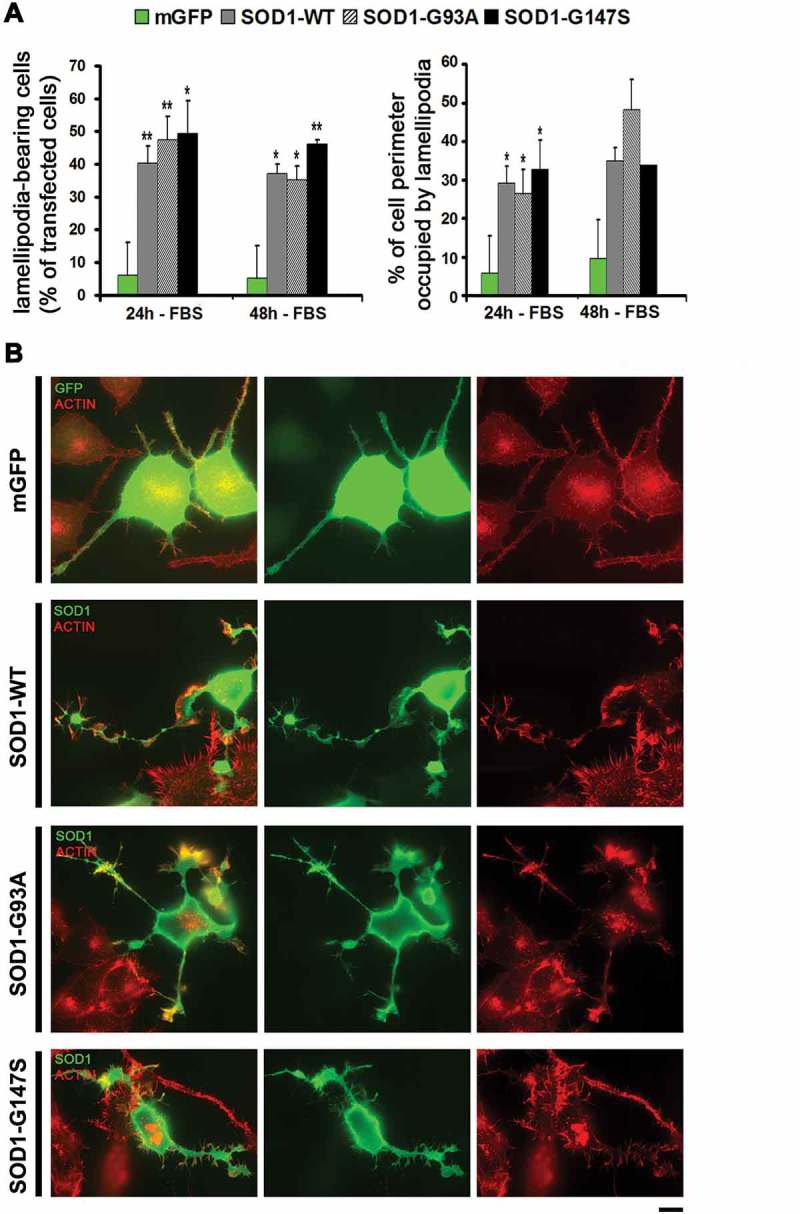
Lamellipodial extension induced by the expression of wild-type SOD1 and ALS-linked SOD1 mutants in N2A cells cultured under differentiated conditions. (A-B) N2A cells transfected with the indicated cDNAs were cultured in serum depleted media (-FBS) for additional 24–48 hours before fixation. (A) The histograms represent the percentage of the transfected cells bearing lamellipodia (n > 120 cells from 3 independent experiments), and the mean percentage of cell perimeter occupied by lamellipodia (n = 60 cells from three independent experiments). Error bars indicate ± s.e.m. P-values (t-test): *P < 0.05, **P < 0.01 and ***P < 0.001. (B) Representative wide-field microscopy of N2A cells transfected with the indicated cDNAs (left), and stained with Rhodamine-conjugated Phalloidin to visualise F-actin in lamellipodia (red), and anti-SOD1 (green). Scale bar: 10 µm.

In order to evaluate the effect of SOD1 expression on membrane dynamics in differentiated N2A cells, 24 hours after transfection cells were cultured for additional 24–48 hrs in serum-deprived media (-FBS) prior to fixation and immunofluorescence staining for F-actin and SOD1 to analyse lamellipodial protrusions in transfected cells (Figure 2).

The quantitative analysis indicated significant higher lamellipodia-inducing activity in N2A cells transfected with SOD1, either WT or mutants, compared to control mGFP transfected cells: upon serum deprivation for 24 or 48 hrs, the percentage of cells with lamellipodia dropped to under 10% in mGFP transfected cells, while it remained high (~40%) in SOD1-transfected cells. Moreover, in these cells, lamellipodia occupied one third of their entire perimeter (Figure 2(A)).

N2A cells expressing mGFP showed a morphology typical of differentiated neuronal cells (Figure 2(B)), characterised by few short and one longer neurite emerging from the cell body, with occasional lamellipodia restricted to their extremity (growth cone). This cell morphology was significantly altered in SOD1 transfected cells: processes of similar length were emerging from the cell body and lamellipodia were not restricted to their growth cones, but rather unusually found all along neurite shafts. These alterations were virtually never observed in mGFP expressing cells, while they were clearly detected also in cells overexpressing all SOD1 constructs. The altered morphology observed in these cells suggest that overstimulation of lamellipodial pathways might be detrimental to neuronal differentiation.

## Discussion

This study is based on data in literature reporting that wild type SOD1 and some ALS-mutated SOD1 forms bind and stabilise the Rho GTPases Rac1 [7]. In glial cells, the authors identified NOX2 as a target of Rac1 activity promoted by SOD1. However, Rac1 activity is mainly involved in the organisation of actin-based lamellipodial protrusions in all the cell types, with pivotal roles in neurons, where these structures are required for axon extension and guidance, formation of axon branches and synaptic structures [9]. We therefore hypothesised that overexpression of SOD1 and ALS-associated mutated SOD1 would promote lamellipodial protrusions in neuronal cells. This hypothesis was tested in N2A cells cultured under undifferentiated and differentiated conditions, and in both cell culture conditions we found that the expression of SOD1 promoted a three-fold increase in lamellipodia, identified by their typical morphology of sheet-like membrane protrusions enriched in F-actin.

The involvement of Rac1 in the SOD1 mediated pathway for lamellipodia is inferred by results obtained in undifferentiated N2A cells. In these cells we found that: i) overexpressed SOD1 stimulated lamellipodia to similar extent than overexpressed Rac1; ii) SOD1-mediated lamellipodia were prevented by inhibition of Rac1 pathways (by coexpression of the dominant negative Rac1 N17 or in cells downregulated for a downstream effector of Rac1, the IRSp53-LIN7 complex); (iii) no further increase in lamellipodia was observed in SOD1 cells coexpressing Rac1, IRSp53 or LIN7.

Collectively these data support the possibility that the increase in lamellipodia observed in the surface of cells overexpressing SOD1 is a consequence of SOD1 activation of Rac1-mediated pathway for lamellipodial extension. Harraz and colleagues [7] have previously shown that wild type SOD1 and mutants L8Q and G10V (both lacking their catalytic activity) bind and activate Rac1, our data suggest that this also occurs for the G93A and G147S mutants. In line with the possibility that also these SOD1 mutants bind and activate Rac1, the similar increase in lamellipodia found in cells overexpressing G93A and G147S is not surprising. However, reduced rather than increased levels of activated Rac1 (Rac1GTP) were found in cell lysates of SH-SY5Y neuroblastoma cells overexpressing SOD1 mutants G93A and H80R [18]. The decrease in activated Rac1 may be due to differences in neuronal cell type, alternatively, the lamellipodia measurement used in our study may better reflect Rac1 activity on the cell surface than total Rac1GTP measured by pull down assays.

The overactivation of lamellipodial pathways was observed either by overexpression of WT or mutated forms of SOD1 and was associated to morphological defects during neuronal differentiation in N2A cells, suggesting a novel mechanism of SOD1-mediated neurotoxicity. Regulatory mechanisms normally preventing toxicity of WT SOD1 at basal expression levels might be unable to operate in conditions of overexpression. In line with this possibility, neuronal tissue abnormalities have been reported in WT SOD1 transgenic mice [19], and abnormally high levels of SOD1 transcripts were reported in spinal cord, brain stem and lymphocytes of sporadic ALS patients [20], although it remains to be investigated whether these conditions are associated to overstimulation of Rac1-mediated pathway for lamellipodia.

## Materials and methods

### Constructs

The constructs GFP-IRSp53, GFP-LIN7A and shRNA LIN7C have been previously described [15,16]; shRNA IRSp53 was purchased from Sigma Aldrich. A 50% reduction of LIN7 was achieved by shRNA LIN7C [16], while a slightly lower downregulation of IRSp53 was detected by Western blot in cells transfected with shRNA IRSp53 (Figure S2). cDNAs for human SOD1 and SOD1G93A were a kind gift of Dr. C. Bendotti [21]. SOD1 G147S was generated by means of Quickchange Site-directed Mutagenesis (Stratagene, CA) polymerase chain reaction (PCR) using the hSOD1 cDNA as the template. The full-length cDNA insert was excised and recloned into the BamHI and NotI sites of the mammalian expression vector pcDNA3 (Invitrogen, Paisley, UK). The absence of unwanted substitution was checked by sequencing.

The GFP construct fused to a membrane localisation sequence (mGFP) was a kind gift from Dr N. Borgese [22]. pCDNA3-Rac1-HA and pCDNA3-Rac1N17 were a kind gift of Dr. G. Scita.

### Cell culture and transfection

Murine neuroblastoma Neuro2A (N2A) cells [23] were grown in DMEM with 10% foetal bovine serum (FBS), 1 mM glutamine and antibiotics. The cell lines were cultured in a 37°C incubator containing 5% CO_2_. Transfections: cDNAs and shRNAs were transiently transfected in N2A cells by using PEI (Invitrogen, Carlsbad, CA), following the manufacturer’s protocol. To maintain the same dosage of transfected SOD1 in all the experiments, SOD1 cDNA has been always cotransfected with control empty vectors or with the cDNA of interest. Twenty-four hours after transfection cells were cultured for additional 24 or 48 hours in media containing 15% FBS (undifferentiated N2A), or in regular media depleted of serum (-FBS) for 24 or 48 h (differentiated cells) before paraformaldehyde fixation.

### Antibodies

The polyclonal rabbit anti-LIN7 antiserum was raised against the histidine-LIN7A fusion protein [15]; the polyclonal rabbit anti-IRSp53 was a kind gift from Dr E. Kim (Korea Advanced Institute of Science and Technology) [24]. Commercial primary antibodies: sheep polyclonal against human SOD1 (Calbiochem, Darmstadt, Germany) and mouse monoclonal anti-HA antibody (Invitrogen, Paisley, UK).

### Immunofluorescence

After being grown and transfected as described, the cells were fixed for 20 min in 4% paraformaldehyde in 0.1 M phosphate buffer (pH 7.4) and permeabilised with 0.5% Triton X-100. Immunostaining with primary antibodies was followed by incubation with Fluorescein isothiocyanate (FITC)/CY5 anti-rabbit/mouse antibodies (Jackson ImmunoResearch). Texas-red Phalloidin was used to stain F-actin (Molecular Probes). Wide-field microscopy was performed with a Zeiss Axioplan inverted phase-contrast microscope (60x objective) connected with an AxioCam HRm CCD camera.

### Image and statistical analysis

For quantification of the percentage of cells–bearing lamellipodia: The percentage was manually quantified using a Zeiss Axioplan inverted phase-contrast microscope (60× objective) connected with an AxioCam HRm CCD camera. Lamellipodia were defined as ruffled plasma membrane processes containing brush-like actin filaments. This definition in no way attributes any functional value to these structures and is purely a reflection of morphological similarity to lamellipodia. For each condition and transfectant, data are the mean ± s.e.m. of 150 cells examined in randomly chosen fields from 3 independent experiments.

For quantification of the percentage of cell perimeter occupied by lamellipodia, wide-field images of transfected N2A cells were analysed with ImageJ as follows: the full perimeter of each cell was measured with the “segmented line” tool. Lamellipodia of the same cells (identified by both the typical ruffled appearance of the plasma membrane and the brush-like appearance of F-actin) were then outlined and measured with the same tool. For each condition and transfectant, data are the mean ± s.e.m. of 60 cells from 3 independent experiments.

All quantitative data are presented as means ± s.e.m.; multiple comparisons among groups were carried out with Student’s t-test using Prism software (GraphPad PrismTM software).

## Supplementary Material

Supplemental Material
