# Supplementary material for: SOD1 stimulates lamellipodial protrusions in Neuro 2A cell lines
Source: Commun Integr Biol. 2018 Aug 9;11(3):1–7. doi: 10.1080/19420889.2018.1486652 (PMC6132423; doi:10.1080/19420889.2018.1486652)
Supplement: Supplemental Material [file kcib-11-03-1486652-s001.zip › Suppl fig caption.docx]

SUPPLEMENTARY FIGURES

Figure S1. Western blot analysis of the level of expression of the indicated SOD1 constructs transiently cotransfected in N2A cells. Twenty-four hours after transfection, total protein extracts were analysed by 12% SDS-PAGE followed by immunoblotting. The blot was probed for SOD1 and actin, the latter as a loading control. SOD1 antibody recognised an upper band corresponding to the expected molecular weight of human SOD1 (hSOD1) and a lower band corresponding to the endogenous murine SOD1 (mSOD1).

Figure S2. Western blot analysis of the level of downregulation of IRSp53 in cells transiently transfected with shRNA IRSp53 (Sigma Aldricht). Total protein extracts from control and silenced N2A cells were probed for IRSp53 and TOM20, the latter used as a loading control. The histogram representing the percentage of IRSp53 in silenced cells compared with control was obtained by densitometric quantification of IRSp53 signal normalised to TOM20.
