# Supplementary figures and images for: SOD1 stimulates lamellipodial protrusions in Neuro 2A cell lines
Source: Commun Integr Biol. 2018 Aug 9;11(3):1–7. doi: 10.1080/19420889.2018.1486652 (PMC6132423; doi:10.1080/19420889.2018.1486652)

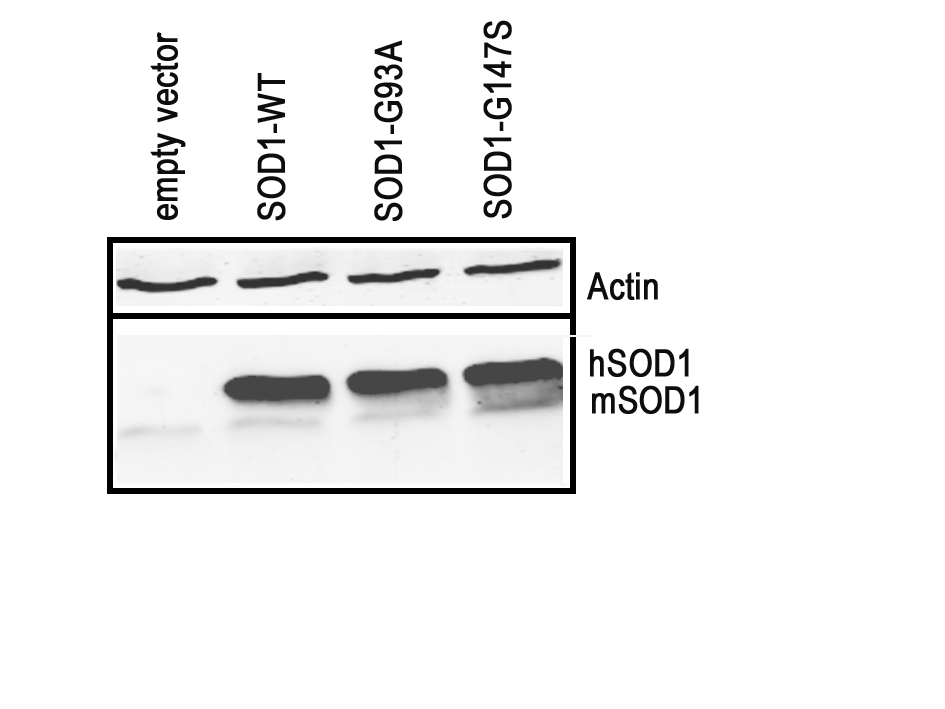

Supplement: Supplemental Material [file kcib-11-03-1486652-s001.zip › S. 1.tif]

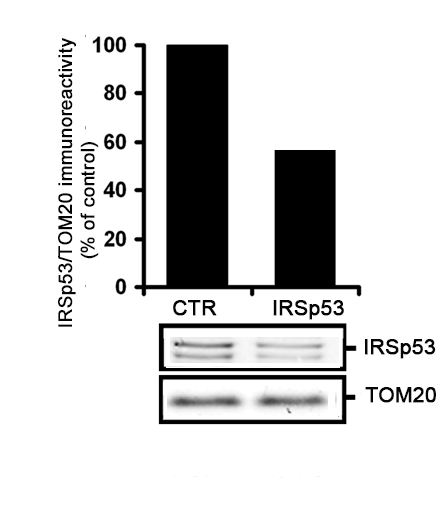

Supplement: Supplemental Material [file kcib-11-03-1486652-s001.zip › suppl 2.tif]
